# Comparative repeatome analysis on *Triatoma infestans* Andean and Non-Andean lineages, main vector of Chagas disease

**DOI:** 10.1371/journal.pone.0181635

**Published:** 2017-07-19

**Authors:** Sebastián Pita, Francisco Panzera, Pablo Mora, Jesús Vela, Ángeles Cuadrado, Antonio Sánchez, Teresa Palomeque, Pedro Lorite

**Affiliations:** 1 Sección Genética Evolutiva, Facultad de Ciencias, Universidad de la República, Montevideo, Uruguay; 2 Departamento de Biología Experimental, Área de Genética, Universidad de Jaén, Jaén, Spain; 3 Department of Cell Biology and Genetics, University of Alcalá de Henares, Alcalá de Henares, Madrid, Spain; Fujian Agriculture and Forestry University, CHINA

## Abstract

*Triatoma infestans* is the most important Chagas disease vector in South America. Two main evolutionary lineages, named Andean and non-Andean, have been recognized by geographical distribution, phenetic and genetic characteristics. One of the main differences is the genomic size, varying over 30% in their haploid DNA content. Here we realize a genome wide analysis to compare the repetitive genome fraction (repeatome) between both lineages in order to identify the main repetitive DNA changes occurred during *T*. *infestans* differentiation process. RepeatExplorer analysis using Illumina reads showed that both lineages exhibit the same amount of non-repeat sequences, and that satellite DNA is by far the major component of repetitive DNA and the main responsible for the genome size differentiation between both lineages. We characterize 42 satellite DNA families, which are virtually all present in both lineages but with different amount in each lineage. Furthermore, chromosomal location of satellite DNA by fluorescence *in situ* hybridization showed that genomic variations in *T*. *infestans* are mainly due to satellite DNA families located on the heterochromatic regions. The results also show that many satDNA families are located on the euchromatic regions of the chromosomes.

## Introduction

*Triatoma infestans*, hemipteran insect of the subfamily Triatominae, is the most important vector of the protozoan *Trypanosoma cruzi*, the causative agent of Chagas disease. Until 1990s, *T*. *infestans* had a wide geographical distribution occupying more than 6 million km^2^ in seven South American countries, and was responsible for well over half of the 18 million people affected by Chagas disease [1]. This species included two main evolutionary lineages, named Andean and non-Andean, recognized by genetic [2,3] and phenetic characteristics [4,5]. These lineages present a clearly differentiated geographic distribution. Andean lineage is dispersed in high altitude valleys of Bolivia and Peru (above 1700 meters above sea level, masl) and also in lower altitudes in Southern Peru. Non-Andean lineage was found in lowland regions (0 to 1400 masl) in Argentina, Brazil, Chile, Paraguay, Uruguay and Bolivia (Chaco region) and also at higher altitudes in some localities of Argentina. Since the 1990s until now, control interventions by multinational initiatives coordinated by the Pan American Health Organization/Word Health Organization (PAHO/WHO), mainly based in houses spraying with pyrethroid insecticides have reduced more than 80% of the original *T*. *infestans* distribution occupying less than 1 million of km^2^ [1]. Non-Andean lineage was virtually eliminated from Brazil, Chile, Uruguay, and in substantial areas of Argentina, Bolivia and Paraguay, persisting only in the Gran Chaco region (southern Bolivia, north-western Paraguay and northern Argentina). On the contrary, the Andean lineage still persists in many localities of Bolivia, probably due to the frequent occurrence of pyrethroid resistant populations [6]. The drastic reduction of the non-Andean group in most of its distribution suggests that *T*. *infestans* lineages have different genetic backgrounds in relation to their susceptibility to pyrethroid insecticides.

The main genetic difference between both lineages is their nuclear DNA content, varying from 1.487 Gbp (non-Andean) to 1.936 Gbp (Andean) per haploid genome, without modifications in their chromosome number (2n = 22). This variation in the genome size has been related with differences in the C-heterochromatin amount [7,8]. Non-Andean group bear C-heterochromatin on 2 to 4 autosomal pairs plus on the Y chromosome, while Andean group presents heterochromatin on 6 to 9 pairs and on both sex chromosomes (XY) [9,10]. Both lineages also differ in the number and chromosomal location of other class of repetitive DNA: the major ribosomal clusters. Non-Andean lineage always exhibits one rDNA locus in the X chromosome, while Andean lineage is polymorphic in number (1–4 loci) and position (on 1, 2 or 3 autosomal pairs and the X chromosome) [10].

Repetitive DNA component is not only often the largest component of the genomes, but it is also essential for different genomic functions. The repetitive DNA distribution, mainly the heterochromatin, establishes a particular nuclear architecture, which can determine distinct transmission and expression properties even with the same coding sequences [11]. So changes in the composition and location of repeated DNA would have a very important role in the evolutionary diversification of the species [12]. In disease vectors, including *T*. *infestans*, it has been suggested that heterochromatin organization, histone modifications, and nuclear architecture can play important roles to determine phenotypes with different vectorial capacity [13]. Probably in *T*. *infestans* lineages, the striking differences in the genome size, C-heterochromatin amount and ribosomal genes location are involved together with other genomic changes, affecting differentially the susceptibility to pyrethroid insecticides as observed in both lineages.

Next-generation sequencing (NGS) techniques are powerful tools that allow carry out a global analysis of the repetitive sequences that form part of a genome [14–16]. The repetitive DNA fraction of a genome, named “repeatome” by Maumus and Quesneville [17], has been frequently omitted or superficially analyzed in most genome-wide analyses [18], probably by their non-coding nature or by the difficulty of the repetitive DNAs assembly. However, there are numerous studies showing the fundamental role of the repetitive sequences in the genome conservation and evolution [19].

Despite *T*. *infestans* medical and social relevance, the research on the nature of its repetitive sequences is very scarce. Using reassociation kinetics (C_0_t) followed by cloning and sequencing, three satellite DNA (satDNA) families have been isolated and determined their chromosome location only in Andean *T*. *infestans* [20]. In this paper NGS techniques are applied in both *T*. *infestans* lineages for first time, in order to characterize and compare the repeat sequences composition of both genomes. The objective was to identify the main repetitive DNA changes occurred during *T*. *infestans* differentiation process. Since results showed that satDNA is the main responsible for the genome size differences between lineages, a thorough analysis of this kind of repetitive DNA was carried out, including the chromosomal location determination for the eleven most abundant satDNA families by fluorescence *in situ* hybridization (FISH).

## Material and methods

### Samples

*Triatoma infestans* samples for DNA sequencing from Tacuarembó (Uruguay) and Tres Estacas (Argentina) were selected for the non-Andean lineage (1 male individual each) and a Cochabamba (Bolivia) sample was used for the Andean lineage (1 male individual). The membership of the selected individuals to each lineage was previously confirmed by C-banding and FISH localization of ribosomal clusters following [10]. Chromosome preparations for FISH analyses were obtained from several samples from Argentina and Uruguay (non-Andean lineage), and Bolivia (several localities from Cochabamba and La Paz) (Andean lineage).

No specific permissions were required for insect collections performed in this work, and did not involve endangered or protected species.

### DNA sequencing and data analyses

For each sample, approximately 3 μg of genomic DNA were employed in Illumina® Hiseq™ 2000 paired-end sequencing–with 100 bp reads. 2.4 Gbp of sequences were obtained from Uruguay sample (coverage 1.35x), 1.9 Gbp from Argentina sample (1.7x) and 4.4 Gbp from the Bolivian sample (2.3x). Graph-based clustering analyses on the three samples were first carried on separately using RepeatExplorer, implemented within the Galaxy environment (http://repeatexplorer.org/) [14,21]. In addition, we performed a combined data set analysis, since facilitates finding shared repeat families of unequal abundance among species, while the individual genome screening facilitates the detection of species specific repeat families [14]. Sequences corresponding to mitochondrial DNA were eliminated for the repeat analyses. The genome proportion for each cluster was calculated as the reads percentage. Additionally, repeat type identification was corroborated by sequence-similarity searches of the assembled contigs against GenBank using BlastN and BlastX (http://www.ncbi.nlm.nih.gov/) and Repbase using CENSOR (http://www.girinst.org/). To identify potential satellite repeats, contigs were analyzed using Dotmatcher (available on http://emboss.bioinformatics.nl/cgi-bin/emboss/dotmatcher/). Estimates of evolutionary divergence between sequences were conducted with MEGA 6 using p-distance.

### Cytogenetic mapping

Meiotic chromosome preparations for FISH analyses were obtained from male gonads. Testes were removed from living adult insects, fixed in an ethanol–glacial acetic acid mixture (3:1) and stored at -20°C. Squashes were made in a 50% acetic acid drop, coverslips were removed after freezing in liquid nitrogen and the slides were air dried and then stored at 4°C.

The consensus sequence of each satDNA family (S1 Table) was used to designed one or two oligonucleotides (S2 Table). SatDNA families with monomer longer than 85 bp were amplified by PCR in 25 μl reaction mixtures containing 50 ng of genomic DNA, 0.5 mM dNTPs, 50 pmol of the primers and 1 U of Taq polymerase (Bioline). The PCR program used was 2 min at 92°C and 35 cycles: 20 sec at 92°C, 60 sec at 51°C, 2 min at 72°C, with a final extension of 5 min at 72°C. PCR products were analyzed by electrophoresis in agarose gels and amplified bands were eluted from the gel and inserted into the pGEMT-Easy vector (Promega). Recombinant plasmids were sequenced. FISH using plasmids probes were performed as described by Palomeque *et al*. [22]. Probe labeling was carried on with biotin-16-dUTP (Roche®) using a Nick Translation Kit (Roche®), following manufacturer's instructions. Hybridization solutions were prepared to a final concentration of 5 ng probe/ml in 50% formamide. Hybridization was conducted at 37°C overnight. For satDNA families with monomer shorter than 85 bp (and for TinfSat06-181), one or two oligonucleotides based on the most conserved regions were directly labeled with biotin-16-dUTP using terminal transferase (Roche®) and following the instructions of the supplier. Hybridization solutions were prepared to a final concentration of 200 pmol probe/ml in 50% formamide. Hybridization was also conducted at 37°C overnight. Fluorescence immunological detection was performed using the avidin-FITC/ anti-avidin-biotin system with two rounds of amplification. Slides were mounted with Vectashield (Vector®). DAPI in the antifade solution was used to counterstain the chromosomes.

Images capture and analysis were made using a BX51 Olympus® fluorescence microscope equipped with a CCD camera (Olympus® DP70) and processed with Adobe® Photoshop® software.

## Results

### *Triatoma infestans* repeatome composition

**Fig 1:**
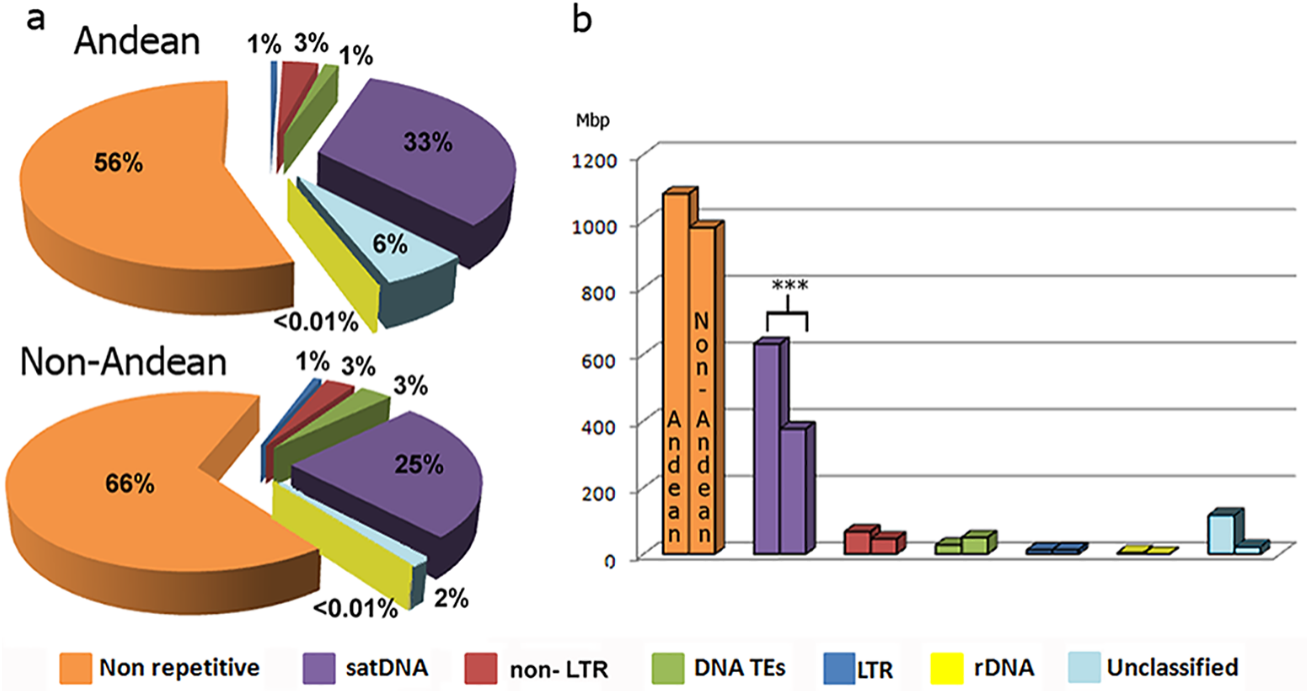
*T*. *infestans* from Andean and non-Andean lineages repeatomes. (a) Pie charts showing total percentages of each category in the genome, including repetitive and non repetitive DNA. (b) Comparative charts showing the amount of each category expressed in mega base pairs (Mbp) per haploid genome. Chi-square test significant differences are depicted with asterisks (p< 0.001).

Graph-based clustering was used to characterize, quantify and compare highly repetitive DNA sequences in Andean and non-Andean *T*. *infestans* genomes. In the Andean sample (Bolivia) the clustering of 199,646 reads produced 164 clusters. While the two non-Andean samples (Argentina and Uruguay individuals) retrieved clustering of 270,604 and 274,852 reads which produced 131 and 136 clusters respectively, excluding clusters matching to mitochondrial DNA. Since each repetitive DNA category (see later) percentages were practically the same in both non-Andean individuals; data about non-Andean lineage is an average between both analyzed individuals. All repetitive clusters (repeatome) corresponded to 44% and 34% of the total genome in Andean and non-Andean lineages respectively. Clusters could be classified into 6 categories or groups: Long Terminal Repeats (LTR), non Long Terminal Repeats (non-LTR), class II elements or DNA transposons (DNA TEs), satellite DNA (satDNA), ribosomal DNA (rDNA) and undetermined repeats (unclassified) (Fig 1). This last group includes clusters that could not be assigned to any category. Considering the haploid genome, frequency and amount in Mega base pairs (Mbp) of each category are shown in Fig 1B. Non-repetitive sequences represent 56% and 66% in each genome, equivalents to 1081.92 and 980.31 Mbp per haploid genome, Andean and non-Andean lineages respectively (Fig 1A and 1B). When we developed the combined data analysis on RepeatExplorer among the three samples, the same results were retrieved for the Andean lineage. However, it was successful to identify several satDNA families also present in the non-Andean lineage. These families are depicted on the S1 Table as <0.1% abundance on the genome.

SatDNA is by far the main component of the repetitive DNA in both lineages, being the 33% in the Andean and the 25% in the non-Andean genomes (Fig 1A). The second most common fraction is composed of the non-LTR sequences with a 3% in both genomes (Fig 1A). No substantial variations were observed in richness and kinds of non-LTR between both lineages. Most non-LTR clusters belong to the LOA clade, but other clades detected were: I, CRE, R1, R2, jockey, L2 and Penelope, which are not strictly non-LTR elements but here we considered together to simplify. DNA TEs represents the 1% and 3% in Andean and non-Andean genomes respectively. Within them, *mariners*-like elements constituted the half of them, while helitrons represented about 10% of DNA TEs. Other families found in low percentages were hAT, mutator and PIF-Harbinger. Also a small fraction of DNA TEs were not classified into any family. Lastly LTR elements were scare, representing the 1% of both genomes. Most of them are represented by Gypsy elements, followed by BEL/Pao and copia elements.

We compared by Chi-square test the amount of all genome fractions between both lineages, including non-repetitive DNA. Only satDNA have statistically significant differences (p<0.001). Hence, the genome content difference between both lineages is mainly due to the quantity of satDNA fraction (Fig 1B).

### *Triatoma infestans* satellitome composition

RepeatExplorer analysis using the Illumina reads from the Andean and non-Andean samples resulted in the characterization of 42 different satDNA families (S1 Table, Acc. Numbers: KY242402-KY242443). The nomenclature used for each satDNA family was the proposed by Ruiz-Ruano *et al*. [16], which include: species name abbreviation, a number in decreasing abundance order and the repeat unit size. Since there are differences in the amount of each satDNA family between both lineages, the Andean lineage was chosen to designate satDNA family names. So the most abundant family in the Andean lineage was called as TinfSat01-33, the second one as TinfSat02-79 and so on.

Thirty-four of the 42 satDNA families have been found in both lineages. The sequence of each satDNA is conserved between both lineages, with the same consensus sequence. One family was only detected in the Andean lineage and seven only in the non-Andean lineage (S1 Table). The five most frequent satDNA families represent the 94% and the 83% of the total satDNA in Andean and non-Andean lineages respectively. However, the amount of each satDNA family is highly variable between them. For instance, TinfSat01-33 is the main family in the Andean sample (11.77% of the genome), but not in the non-Andean sample (3.55%). In the non-Andean genome the main family is TinfSat02-79, representing a 10.03% of the genome (S1 Table).

There is a great variation in relation with the repeat unit length (4 to 1,000 bp) (S1 Table). However, most of the satDNAs (34 of the 42 families) have a repeat unit below 120 bp. The A+T content in the satDNA families ranging between 55.0 and 81.3%, with the exception of the TinfSat37-314 family (44.6%).

Most of the different satDNA families seem to be non-related. The existence of satDNAs with similar monomer length could indicate that they are related, such as TinfSat37-314 and TinfSat38-315. Consensus sequence alignment of both satDNAs shows that they have a similarity of 58.5% that could suggest a common origin. Regions with similarity have been also found between satDNA families with different monomer length. So, TinfSat22-64 sequence contains a 23 bp region with a 91% of similarity with the TinfSat09-113 monomer. A more complex relationship could exist between other families, as TinfSat04-1000 and TinfSat42-112, which despite the size differences of the repeat units, share a 104 bp region with an 84% of similarity.

The existence of higher order repeats (HOR) has been observed for other satDNA families, as TinfSat09-113. The analysis of the sequence of this satDNA shows that contains four internal subrepeats (S1 Fig). This satDNA would therefore has originated from a 25 bp satDNA that has diverged during evolution by duplications and probably by short insertions. The existence of HOR has been also observed for other satDNA families, specifically for TinfSat03-4 and TinfSat05-4. Both satDNAs have a 4 bp repeat unit (GATA and CATA respectively) and the majority of the analyzed contigs are formed by a tandem repeat of these simple sequences. However, for TinfSat03-4 it is possible to detect other contigs with new repeat units probably generated from these simple repetitions. So, in Bolivia and Argentina individuals there are tandem repeats with the (GATAGTTA)_n_ sequence and in Uruguay with (GATAGATTA)_n_ or (GATAGGTA). For the TinfSat05-4 family a new repeat unit has been detected in Andean as well in non-Andean individuals, (CAATACATACATACATA)_n_.

An interesting satDNA family is TinfSat04-1000. This satellite DNA contains an internal microsatellite (CA)_6-20_, so the length of the monomeric repeat is variable in function of the microsatellite repeats number. A similar organization has been recently described for a satDNA family in *Locusta migratoria*, with different size variants due to the number of repeats of a GA microsatellite [16].

### Main satDNA families’ chromosome location

**Fig 2:**
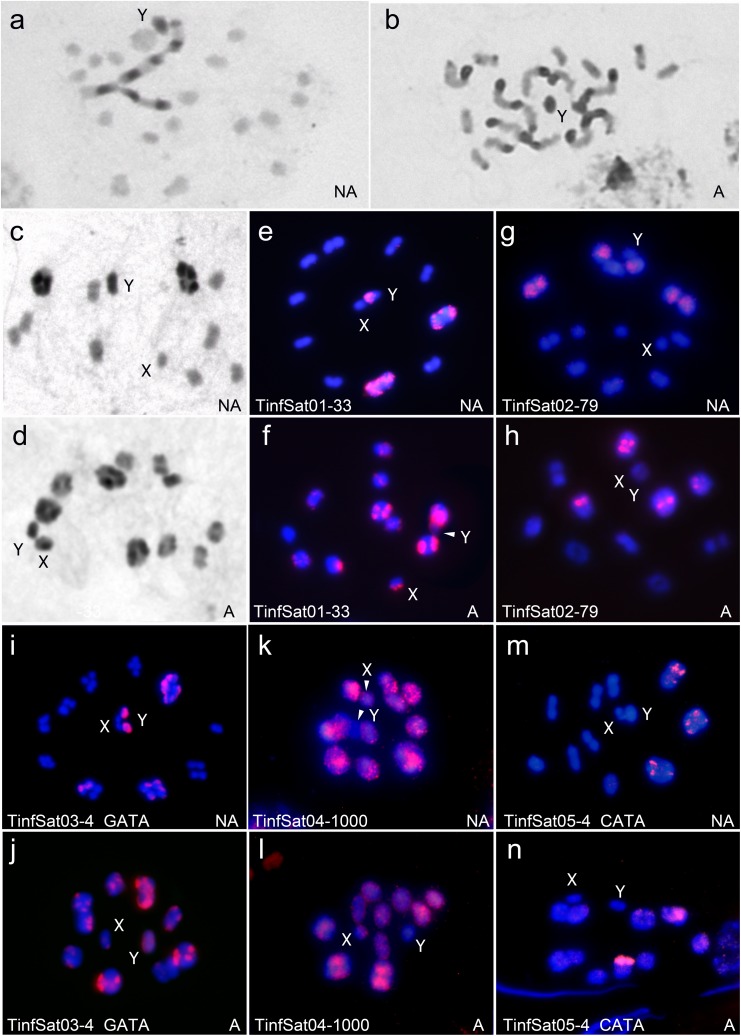
C-banding patterns and satDNA families’ hybridization observed in *Triatoma infestans* in non-Andean (NA) and Andean (A) lineages (2n = 20 autosomes plus XY in males). **Abbreviations: first meiotic metaphase (MI), anaphase I (AI) and second meiotic metaphase (MII).** (a) C-banding. NA lineage (spermatogonial mitotic metaphase): four autosomes and the Y chromosome present C-heterochromatic regions. (b) C-banding. A lineage (spermatogonial metaphase): almost all chromosomes present C-bands in addition to the Y chromosome. (c) C-banding. NA lineage (MI): only two bivalents and the Y chromosome present heterochromatic regions. The X chromosome is euchromatic. (d) C-banding. A lineage (MI): heterochromatic Y and almost all bivalents present C-bands. The X chromosome also shows a heterochromatic block. (e) TinfSat01-33. NA lineage (MI): hybridization signals on heterochromatic regions of two bivalents and the Y chromosome almost entirely. (f) TinfSat01-33. A lineage (MI): hybridization signals on nine bivalents, the Y chromosome and a region of the X chromosome. (g) TinfSat02-79. NA lineage (early AI): Hybridization signals are restricted to heterochromatic regions of three autosomal bivalents. The heterochromatic Y chromosome appears labeled free. (h) TinfSat02-79. A lineage (MI): Hybridization signals on four bivalents. X and Y chromosomes lack hybridization signals. (i) TinfSat03-4 (GATA)_n_ repeats. NA lineage (early AI): hybridization signals on heterochromatic regions of three bivalents and the Y chromosome. (j) TinfSat03-4 (GATA)_n_ repeats. A lineage (MI): hybridization signals on heterochromatic regions of nine bivalents and both sex chromosomes (X and Y). (k) TinfSat04-1000. NA lineage (MI): hybridization signals on euchromatic regions of all bivalents (10) and the X chromosome. The heterochromatic Y chromosome did not display labeling. (l) TinfSat04-1000. A lineage (MI): hybridization signals on euchromatic regions of all bivalents (10) and the euchromatic region of the X chromosome. The heterochromatic region of the autosomes, the X and the Y chromosome did not display labeling. (m) TinfSat05-4 (CATA)_n_ repeats. NA lineage (MI): hybridization signals on the heterochromatic regions of three bivalents. (n) TinfSat05-4 (CATA)_n_ repeats. A lineage (MI): hybridization signals on the heterochromatic regions of four bivalents and weak signals in other five bivalents.

**Fig 3:**
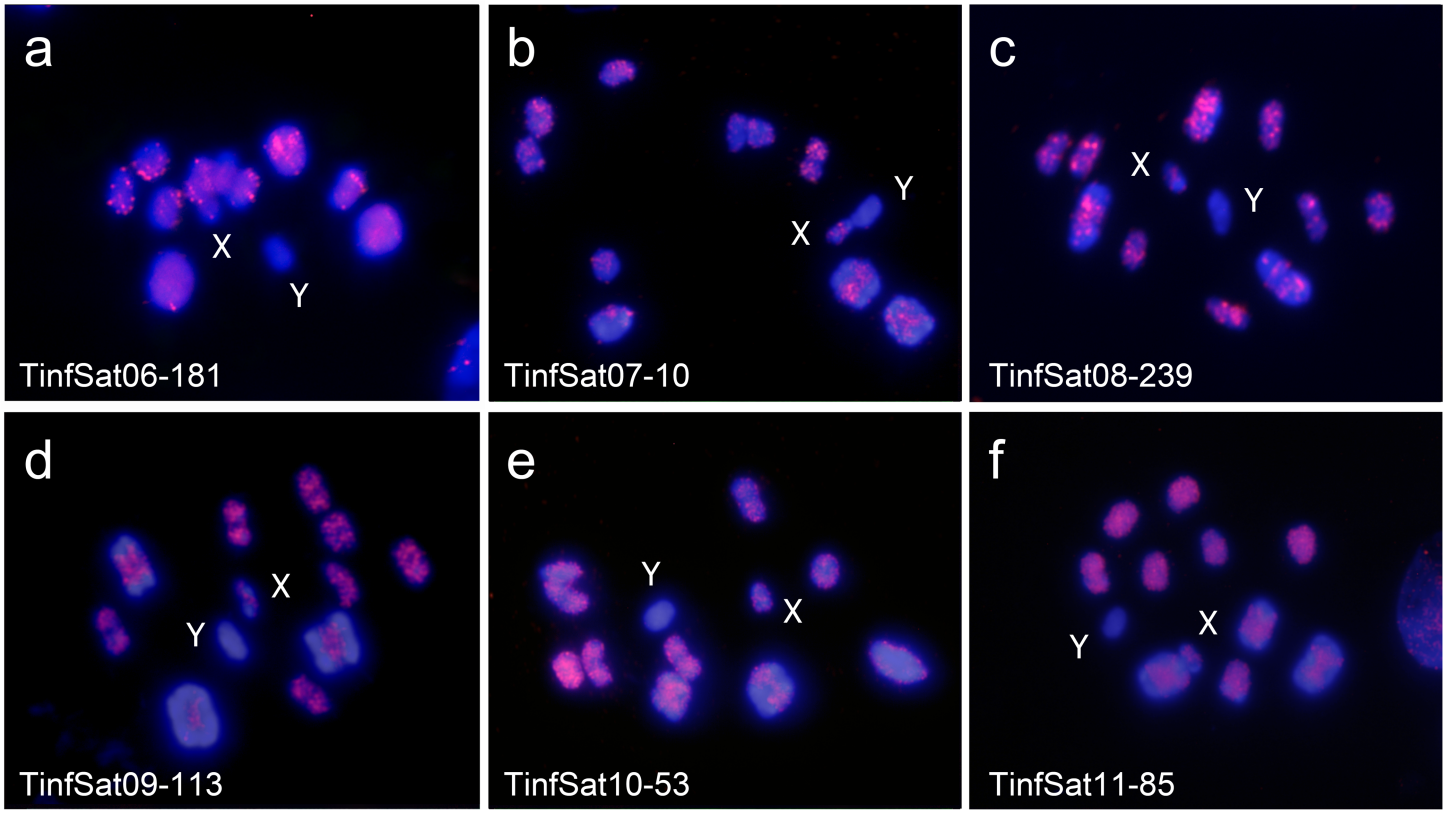
SatDNA families’ hybridization observed in *Triatoma infestans* non-Andean lineage. (a) TinfSat06-181; (b) TinfSat07-10; (c) TinfSat08-239; (d) TinfSat09-113; (e) TinfSat10-53; (f) TinfSat11-85: All images in first meiotic metaphase. Hybridization signals on euchromatic regions of all autosomes and X chromosome, while that the autosomal heterochromatic regions and the Y chromosome appeared labeled free.

We have located by FISH eleven satDNA families that were shared between both lineages and that represent more than 0.03% in each genome. Their richness in the genome and chromosome location of each one in both lineages is showed in Table 1, Fig 2 and Fig 3. We have found that the different satDNA families could be located on the heterochromatic regions as well as on euchromatic regions. The hybridization patterns between Andean and non-Andean lineages are very different for satDNAs located on the heterochromatic regions. As above commented, non-Andean lineage bears C-heterochromatin in less autosome pairs than the Andean lineage (Fig 2A, 2B, 2C and 2D). As consequence the number of autosomal bivalents that show positive hybridization is greater in the Andean lineage than in the non-Andean lineage (compare Fig 2E with 2F, 2G, 2H, 2I and 2J or 2M and 2N). The hybridization patterns for satDNAs located in the euchromatic regions are more similar between both lineages, since hybridization signals are spread over all chromosomes, less the heterochromatic Y chromosome. We recognized three satDNA family types according to their chromosomal location: (a) satDNA families (TinfSat01-33 and TinfSat03-4) located on C-heterochromatic regions of the autosomes and both sex chromosomes (Fig 2E, 2F, 2I and 2J). (b) satDNA families (TinfSat02-79 and TinfSat05-4) located on the C-heterochromatic regions of the autosomes but not on the Y chromosome (Fig 2G, 2H, 2M and 2N) (c) satDNA families located on the euchromatin regions of the autosomes and the X chromosome (TinfSat04-1000; TinfSat06-180; TinfSat07-10; TinfSat08-239; TinfSat09-113; TinfSat10-53; TinfSat11-85) (Figs 2K, 2L and 3A–3F). As the X chromosome in non-Andean lineage lacks of heterochromatin, no hybridization signals were observed with the satDNA families belonging to the first two types.

**Table 1:** *Triatoma infestans* satDNA families’ quantification in Andean and non-Andean lineages. : All data are expressed in relation to the haploid genome. Abbreviations: A = Autosomes, E = euchromatin, H = heterochromatin, X = euchromatic X chromosome, X* = heterochromatic X chromosome in Andean lineage, Y = Y chromosome. Nucleotide motifs of the 11 satDNA families are included in S1 Table.

SatDNA family	Andean (Mbp)	Non-Andean (Mbp)	Chromosome Localization
TinfSat01-33	228.78	37.76	H: A+X*+Y
TinfSat02-79	174.83	149.63	H: A
TinfSat03-4	88.87	66.29	H: A+X*+Y
TinfSat04-1000	82.86	40.52	E: A+X
TinfSat05-4	27.56	14.15	H: A
TinfSat06-181	4.66	1.42	E: A+X
TinfSat07-10	2.53	0.15	E: A+X
TinfSat08-239	1.55	1.05	E: A+X
TinfSat09-113	1.36	3.35	E: A+X
TinfSat10-53	0.97	0.45	E: A+X
TinfSat11-85	0.58	0.45	E: A+X
TOTAL Mbp	614.53	315.19	

## Discussion

Total repetitive DNA content (40% mean between both lineages) is in the same range reported for other hemipteran species, as in the pest rice *Nilaparvata lugens* (48.6%) [23] or the pea aphid *Acyrthosiphon pisum* (33.3%) [24]. However, *T*. *infestans* repeatomes in both lineages are mostly composed by satDNA, representing about the 30% of the total genome in comparison with the 6.4% of *N*. *lugens*. In this last species as well in the pea aphid, the main repetitive fraction consists in transposable elements (TEs) (39% and 38% respectively) unlike the 5–7% observed in *T*. *infestans*. Other non-hemipteran insects with holocentric chromosomes as the lepidopteran *Bombyx mori* has in its genome a 43.6% of highly repetitive sequences, also mainly composed by TEs [25].

*T*. *infestans* is divided in two main lineages, Andean and non-Andean, with significant differences in their genome size (1.936 Gbp and 1.487 Gbp per haploid genome, respectively) [7,8]. Our results showed that both lineages have similar quantity of non-repetitive sequences, without statistically significant differences (Fig 1). The results obtained with RepeatExplorer showed that *T*. *infestans* repeatomes in both lineages are mostly composed by satDNA. Furthermore, Andean lineage presents almost twice more satDNA than the non-Andean lineage (630.78 Mbp and 376.05 Mbp per haploid genome, respectively) (Fig 1B), being the main responsible for the genome size differentiation between both lineages. The analysis of this genome fraction shows that satDNA families are conserved between lineages. Thirty-four of the 42 satDNA families described were found in both lineages, one family was only detected in Andean lineage and seven only in non-Andean lineage (S1 Table). One possible hypothesis is that the differentiation of both lineages has been accompanied also by changes in the satDNA composition. Another hypothesis is that all satDNA families are present in both lineages but some of them have not been detected by the RepeatExplorer analyses. The obtained data support the second hypothesis. First, the exclusive families present very low frequencies and second, one of these families, TinfSat39-5, is the telomeric repeat found in several triatomine species [26]. It is hardly unlikely that telomeric repeats are absent in Andean lineage. So, although satDNA families are the same in both lineages, the reason for such a huge difference in DNA content lies on the amount of these same families in each sample (Table 1, S1 Table). This is in accordance with the "library" hypothesis, which predicts that related species share an ancestral set of different conserved satDNA families, which may be differentially amplified in each species due to stochastic mechanisms of concerted evolution [27,28]. In fact, most of the difference between *T*. *infestans* lineages is just due to five satDNA families (TinfSat01 to 05), especially the TinfSat01-33 that varies more than six times between lineages (Table 1). FISH results on both *T*. *infestans* lineages showed four of these families located on the heterochromatin (Table 1, Fig 2). Three of them were previously reported on heterochromatic chromosomes only in Andean lineage by Bardella *et al*. [20]. Their results show that the TinfSat01-33 satDNA is located in the heterochromatic regions in four bivalents and on the X chromosome. The Andean individuals analyzed in this work show hybridization in a greater number of bivalents (Fig 2F). However in the non-Andean lineage, where the number of autosomes with heterochromatic regions is lower, we found hybridization signals only in two bivalents (Fig 2E). We did not find hybridization on the X chromosome; probably due to the fact that the X chromosome lacks heterochromatic regions in non-Andean lineages. A similar pattern were observed for the TinfSat02-79 family, that was present in four bivalents in individuals from Andean regions (Fig 2H) and only in three bivalents in non-Andean lineages (Fig 2G). Similar results are also obtained with the TinfSat03-4, were the number of bivalents showing hybridization signals is greater in Andean individuals (Fig 2I) than in non-Andean individuals (Fig 2J). These three satDNA families constitute 984.95 Mbp in the Andean lineage genome and only 507.35 Mbp in the non-Andean lineage genome (Table 1). Hence, genome size differentiation is due to the satDNA, mainly by the higher amount of satDNA families located on heterochromatic regions. Similar conclusion was previously suggested regarding the variation in the number of chromosomes bearing C-bands [9,10].

Previous GISH (genomic *in situ* hybridization) results reported that the heterochromatic Y chromosome is constituted by highly repeated sequences, which are conserved among several species of the Triatomini tribe [29]. Present results show that *T*. *infestans* Y chromosome harbors two satDNA families: TinfSat01-33 and TinfSat03-4 (GATA repeats). However, unpublished results of our group on other *Triatoma* species have demonstrated that GATA repeat is present on the Y chromosome but not the TinfSat01-33 family. So, GATA repeats would be the only satDNA family that is shared on the Y chromosome in all species of Triatomini tribe. This (GATA)_n_ motif is reported to be extended on Y or W chromosomes of several vertebrates, including human, mouse and snakes, and invertebrates species [30–32]. Furthermore, (GATA)_n_ motif (included in Bkm repeats with (GACA)_n_ motifs) could be required for sex determination and differentiation as well as to participate in the higher order chromatin organization and function, particularly in the formation of heterochromatin [33–35].

Although it is assumed that satDNA is mainly located on the heterochromatin, our data show that there is an important satDNA fraction located on euchromatic chromosomal regions. In *T*. *infestans*, FISH reveals that seven out of eleven analyzed DNA families are located on euchromatic regions of the autosomes and the X chromosome (Figs 2K, 2L and 3A–3F). These results agree with those obtained by microdissection of the X chromosomes, where repeat sequences of these chromosomes were also located on the autosomal euchromatic regions in several triatomine species, including *T*. *infestans* [36]. SatDNA characterization by genome sequencing suggests that their presence on the euchromatic regions is not uncommon, as has been reported in some insect species as *Tribolium castaneum* [37] and *Locusta migratoria* [16], as well as in plant species [38]. For these satDNA families it is probable that classical techniques for satDNA isolation fail due to their lower amount in comparison with the families located in the heterochromatin.

Unfortunately, satDNA comparison with the only Triatominae sequenced species, *Rhodnius prolixus*, is not possible, since data about satDNA were not mentioned at all. For other repeat sequences as TEs (including LTR, non-LTR and class II) the total amount is very similar, being the 6% in *R*. *prolixus* genome [39,40] and 5–7% in *T*. *infestans*.

Although the possible function of the repetitive DNA is still controversial, the satellite DNA has been related to the reproductive isolation and therefore with the appearance of new species [12]. Its importance in genome integrity and in karyotypic evolution has also been highlighted [19]. The chromosome number in Triatominae is highly conserved, indicating that species differentiation seems not to be accompanied by chromosome rearrangements which alter the chromosome number. Our results show that the main genomic change between both lineages is the variation in the amount of satellite DNA. These changes in the genome composition or organization could be related with the species diversification in Triatominae or other animal species groups with low karyotypic variation.

## Supporting information

S1 Table SatDNA families’ complete data of the three sequenced genomes, including genome abundance (%), A+T content (%) and consensus sequences. (DOCX)

S2 Table Used primers for satDNA molecular and cytogenetic analyses. (DOCX)

S1 Fig Aligment of the four regions of the consensus monomeric unit of the TinfSat09-113 satDNA showing internal similarities that could suggest that this satDNA is really a HOR with four subrepeats. (DOCX)
