# Supplementary material for: Comparative repeatome analysis on Triatoma infestans Andean and Non-Andean lineages, main vector of Chagas disease
Source: PLoS One. 2017 Jul 19;12(7):e0181635. doi: 10.1371/journal.pone.0181635 (PMC5517068; doi:10.1371/journal.pone.0181635)
Supplement: S1 Table — (DOCX) [file pone.0181635.s001.docx]

**S1Table**: SatDNA families’ complete data of the three sequenced genomes.including genome abundance (%).A+T content (%) and consensus sequences.

| **SatDNA Family** | **Andean** | **Non-Andean** | **A+T** | **Consensus sequences** | **Acc. number** |
| --- | --- | --- | --- | --- | --- |
| **TinfSat01-33** | 11.77 | 3.54 | 66.7 | TTTCCATAAGTCTATTACTTCGTAATTACTGCG | KY242431 |
| **TinfSat02-79** | 8.99 | 10.04 | 59.5 | CCCTAAAATCGGCGTTTCAAACGTAAGAGTGAGTTTTCGAGCAACACTCACTTCAGTTGGTTGTAAGGTTCAAGAAAAT | KY242402 |
| **TinfSat03-4** | 4.57 | 4.48 | 74.6 | GATA  (GATAGTTA)  (GATAGATTA)  (GATAGGTA) | KY242432 |
| **TinfSat04-1000** | 2.48 | 4.26 | 66.8 | ATCGTCGGAGCCATTTTTGAGAAAATCGCAAAAAAGTAAAAAAAAACAACTAGTAAAGTGGTACTTCCGGTTGAGGAATTTTGACAGATAACGTACTGTCAGATCCCATGGTGATACCTAAACGGAATATCAAGTTTCAACTTTCTACGGTTTTTCGTTTTTGAGCTATGCTGTTCACATACATACAT-(AC)_6-20_-TTTGCTAAAAACCACTTTTTTGGACTCAGGGGACCTCAAAACGGATATTTCCGGTGAAAACTCGATATCGAAAATTTGACACGATTACAATACTTCCTCTTACTAGGAGTAAGAGAAAGTAAAAAAAAAAAATCTGGTGTGAAACACTCACACAACTTTCTCTTACTCCAGTTCTCAAAATTATAATTGACAAATTTTATAGTTTGTGTACCAAATTTAATGAAATTCGTCGAAAACTGAAAAAAACTGTTCAGTAAAAGCGCACTTCCGGTTTACTAATTTTCTTGAAACTCGGAATTTTGGCCATCTATTTATTTCTAATTAATTTTGTTTGGATGATAGAATTGTATAATCAACTATGATAGCACTTTAGAGAAAAACATAACCCCACCCCTCCCCYAAAAGTGCCCTTAAATGAATTTTCCCAGAGAAATGTTTTAAATAAAAGTTGTAGATCTTTGTATGTGTAGTTTATATAGGAAGTTTCAAGAAAATCGTCGGAGCCATTTTTGAGAAAATCGCGAAAAAGTRRAAAAAATCATTTAGTAAAGTGGTAAAGTAAACGCACTTCCGGTTATCCGATTTTTTTCAAACTCGGAATTTCAACAACATATTTAATTCTAATTAACATTATTTAGATGAYAGAATTGTCTAATAAACTGAAATAGCACTTTAGATGAAAACCTAACCCCACCCCTCCCCCAAAAGTGCCCTTAAATGAATTTTCCCRGAGAAATGTTTCAAATAAAAGTTGTAGCTCTTTGTATGTGTAGTTGATATACCAAGTTTAAAGAAA | KY242403 |
| **TinfSat05-4** | 1.42 | 0.94 | 75 | CATA  (CAATACATACATACATA) | KY242433 |
| **TinfSat06-181** | 0.23 | 0.18 | 75.7 | CGGCTCAAAAACAATATTAAAGTCCTTAGAATGTGTTTAGCTAAAATATTATAAGTGTAAACATAATTTAAACATACATAAAAAAAAACAAAGTCTTTGTTCATAGTATTGGATGATAATATATTTAATGTTTTTAGCTAAATATTATAAAATATTAGCCCTCGTCTTGGATGTCATCTCA | KY242404 |
| **TinfSat07-10** | 0.13 | 0.04 | 57.2 | RCATACTCGK | KY242434 |
| **TinfSat08-239** | 0.08 | 0.07 | 59 | TCCCGCGCGAACCTATTCTAAAAACTAAATTCTCTGAAATAAAAGTAAAAGTAAAGAGTGCCTATTTTTTCAGGCTGACGTAACATTCAACTAACAATTAGGTATCGTGGTAGGGGGCACAAACTCCTACTTTACTATTAACATTTTCCATACCGATATAAAATTTAGCAACTCGACGCGGGACCCGAACTGGGGACCTCCCGCGTTCGAGTCCATGCTCACAACCACTAATGAAATAA | KY242405 |
| **TinfSat09-113** | 0.07 | 0.22 | 81.3 | AGAATGTAGAACTTTGAAAAATGTTAGAATGTAGAATTTTGCGTAATATTAGAATGTATAACTTTGGAAAACATGAATTATTAGAATGTAGAACTTTACAAATACTAATTATT | KY242406 |
| **TinfSat10-53** | 0.05 | 0.03 | 58.5 | AACGGTTTTGGTTATACTATTTTTCCAAACCCGCAATACACGTTTGCCCCTTC | KY242407 |
| **TinfSat11-85** | 0.03 | 0.03 | 70.6 | TAAATGCGTGATTCAGCAATTAACATTCCAAGTTATCAACCACTTTACAGATATTTATAAACCATTTTCCTCTCAAATTGAACAA | KY242408 |
| **TinfSat12-84** | 0.03 | 0.02 | 65.5 | AAAAAACATATGCGAACACATACAGGCGAGAAGCCATATAAATGTAGTGAATGTGATTACAGTTGTACACAAtCTGGAAATCTT | KY242409 |
| **TinfSat13-147** | 0.02 | 0.02 | 58.5 | GTTATCTGGTTGGCTTTTTTCCTGCGTTTCAGATTTTGATTCCTTTTCTGCCGCAGTTTTTGATCCCGCTTGAATGAATGGTGGGATTGGCGGAACATCGTCTATTTCAGATATCGATTTGTCAGCATCTTCACCTTCATTACTTGA | KY242410 |
| **TinfSat14-147** | 0.02 | 0.03 | 63.9 | CCAAGAAAGTTCATTTTATGGCGTTTTGGGTGGGGGGTAAGAGGCAAGCTTTTCCCAAAATAGTGGTTTTTATTGTTTAGATTCTCCCACTGATGTAAAATTGAAGACAACTAAACATCTAGTGAGAGAATTAGACTTTTGAAAGTT | KY242411 |
| **TinfSat15-99** | 0.02 | 0.01 | 68.7 | TACGTATTGCGTCATACCGTGCAACATGACATGTCTCAACATGTTCATTTTAATTTATTTCTTACTCTTGCTAAAACATCGAAAAAAATACTAGATTTG | KY242412 |
| **TinfSat16-49** | 0.02 | <0.01 | 73.5 | GAAGTTGTACAATAGTCAATTAGAATTAACAGAAAATTATGTGAAAGGT | KY242435 |
| **TinfSat17-118** | 0.02 | <0.01 | 60.2 | ATCGCAACACTTCCTTTTACTGCTACAACAGAATATAAACTACATGGTATACACCACTTACACCACATTCAGTGCAGAGGGACCTCATTAGACATGTTTCCTAAATAGCTCAATTCCG | KY242413 |
| **TinfSat18-102** | 0.02 | <0.01 | 62.7 | GTCGCGGAAAAAAGTTCCCAATAAAAGTGACGGCATTTGCATTCACAGTTACTGTAGGACGTTTAGTGAAATCCGTTGCCATTTTTACTAAATAAATCAACT | KY242414 |
| **TinfSat19-104** | 0.02 | 0.01 | 64.5 | GCTTAGTCAGTTCAATTATACATTTAGTTGGCTGAATGGTGAGCAACCTAATGTTTGTATTTATTAGCGAGAATGTGATGATAGGTTAGAGTGTGCTTGTATGG | KY242415 |
| **TinfSat20-46** | 0.02 | <0.01 | 69.6 | TCTACACCAGTACATACCTATCATTTTGTTTGAACTTTGTATACTA | KY242436 |
| **TinfSat21-38** | 0.01 | <0.01 | 63.2 | AAACACTACTTCCCAAACTTGATGTTTCCTTTAGACAC | KY242437 |
| **TinfSat22-64** | 0.01 | <0.01 | 78.1 | AATGTGCAAAATCTAAGTAATATTTATGACAAAGTTCTACATTCTAATACTAATATAAAATGGA | KY242416 |
| **TinfSat23-51** | 0.01 | <0.01 | 66.7 | AGATCTATCGCTATTATTTGTTAATGAACCACCCCAACTACAAATATACAC | KY242417 |
| **TinfSat24-112** | 0.01 | 0.01 | 58 | TCCTGCGATGTTGGGGGGTTCCTCCCCCCGCTCTCTGTAGACATAGTGTAACTACACTATTGGCAGTTATATAGTTTTGTCACAATGAAAATGACTTATCAATTTGATGTTC | KY242418 |
| **TinfSat25-62** | 0.01 | 0.02 | 56.5 | TTTTCTGCGATTTTCTCCATAACGGTGGACTAGAAGTGCTACTTCACTGGTGAAGTTCTTCG | KY242419 |
| **TinfSat26-53** | 0.01 | <0.01 | 62.3 | CAGTAAGAGAAAGTGGGGAAATGTATCACATTCAGTTGGCAAGAACTAAATTG | KY242420 |
| **TinfSat27-47** | 0.01 | 0.01 | 61.7 | TAGGTAGTGCTAACACCAACAGAATAATTAATATTACCCCACCACTC | KY242438 |
| **TinfSat28-46** | 0.01 | <0.01 | 65.2 | ATGTACAGCCGATACAGAAAGTTTAAATAAAGTTGTCGCTCTTTGT | KY242439 |
| **TinfSat29-87** | 0.01 | 0.01 | 64.4 | GTGTACAATGTGTGGCACTGACACAAAAACATTTTTTGGGGAGTTGTAAAGTAAGGTTGCTTTACACTTACCTCTAAAGTATTACTT | KY242421 |
| **TinfSat30-58** | 0.01 | <0.01 | 65.5 | TAATCTGAGTTGGAACTATCCATTGTAGTGAACCACATATTTGTTACTATCAGCGTAT | KY242422 |
| **TinfSat31-71** | 0.01 |  | 70.4 | CTAGACTGTATTTAGTTTGTAAAAATATTTTTGTCTGTTGGTCTTGGCCACGTATATAATTATTCGTTTGT | KY242423 |
| **TinfSat32-52** | 0.01 | 0.01 | 59.6 | CACCTCCTACAGCACTAAGAGAAAGTCGTGTGATTACACATCAGATTTGTAT | KY242424 |
| **TinfSat33-372** | 0.01 | 0.02 | 55.8 | TAGTGCTGCGTTTGTGCCGGCGATTCACCAAATTTCATCGTTTGTGCCGTAAAATTGACAAAGATATTAAGGAAAAACTGTTCGTCGTACTTGAAAACCAAAGGCCATGGCTGCGAGTAACATTTGAATGTGCGCGTGCTCCACTGACCGCCAACGGCCCTTATCAGGGCCAGCAACTAAAATGTACTGGCCCTCGATTTCTAATCAAAATTCTGGAATTTTTTCTTCGCTATTCTTAAGTGTTTCATTTGTGCCAGCGACCCACCAAATTTCAGCGTTTGTGCTGCAAAATTGACAAAGTTATTGAGGCGTCAAGTTAGACTGGCCACCAATTTCTATTCACTTTTTTAAACTTTTCTCTTTGCCAATCGT | KY242425 |
| **TinfSat34-28** | 0.01 | 0.02 | 60.7 | TACACCTACATTATAGTACACCTTCTGG | KY242440 |
| **TinfSat35-35** | 0.01 | 0.01 | 68.6 | ACTCATAGGTAGATGATTTAAAACAAAACACCAGT | KY242441 |
| **TinfSat36-10** | -- | 0.06 | 55 | GAGTATGTMC | KY242442 |
| **TinfSat37-314** | -- | 0.04 | 44.6 | GCATATATATGGCTAGCCAGAGTGGTCGTGGGCTGAAAGCCCGCTTAAAACTGGCTGATTGGGCTACTGAGGTTCAATCTGCTGACTATGCCATCACTGCCGGGGCGGCGGTGCCGGAGAAATCGGCGATTAAGGTCGATAACACCACCTATACGGCAGGTAATGACATGACCGTTAGTGTGACGCTGAAGGATGCGCAGGGTAATGGGGTTACTGGCCAAGCTGCTGCCTTAACGTCCCAGGCGGTAACGGTGGCCAATGCCAGTGAGAAAGACGGTGTCACGTGGATCGATAACGGCGAGGCACCTATAGCC | KY242426 |
| **TinfSat38-315** | -- | 0.03 | 61.6 | ACAATTTATAAGGCCGAGACTGTAGGTGAAAACCTGAAAGCGACTGTAAAGCTTCCAGATTGGAAGGGTACAACTGAATCGGCTAATTACGCAATTACTACCGGTAACCCTGCGTATATGAAGTCAACGATTACGTTAGATAAAGACATCTATCCCGCAAATAGTGATATGAAGGTTACGGTAACCTTGAAAGATTTGTATGGTAATAGCGTAACTGGAAAAGTTGCAGAATTAACTGATGCGGTAGTAACAGTACCAAATGCAAAAATGAAAATAGGTGAAAAATGGAAAGAAAATGAAGCTGGGACCTATACT | KY242427 |
| **TinfSat39-5** | -- | 0.03 | 60 | TTAGG | KY242443 |
| **TinfSat40-73** | -- | 0.02 | 60.3 | CCATTTGGGAAACAAGTCGGGCCACCAATACATAGTGAACTAGTAACAAAACGGAACGTTGAAGATTTTGTAA | KY242430 |
| **TinfSat41-101** | -- | 0.01 | 67.3 | AATATATTACAGTGAACACCTATCCTGCGCTGTACTAATGACAAGTTCGAAATAGATGTCTAACAAAATTGTAAAAGAGCTGGGTCTAAAATTATAGCAAT | KY242428 |
| **TinfSat42-112** | -- | 0.01 | 64.5 | GACACTAAGTTATCTGTCAAAATTCCACAACCGGAACAAACACAACCACAACAAAAACAAAAATCGCAGAAAATTCAAACTTGACATTCTGTTTAGGTATCACCGTGGGTTC | KY242429 |
