# Supplementary material for: Comparative repeatome analysis on Triatoma infestans Andean and Non-Andean lineages, main vector of Chagas disease
Source: PLoS One. 2017 Jul 19;12(7):e0181635. doi: 10.1371/journal.pone.0181635 (PMC5517068; doi:10.1371/journal.pone.0181635)
Supplement: S2 Table — (DOCX) [file pone.0181635.s002.docx]

**S2 Table:** Used primers for satDNA molecular and cytogenetic analyses.

| **SatDNA Family** | **Oligonucleotides** |  |
| --- | --- | --- |
| **TinfSat01-33** | TinfSat01-33  5´- TTTCCATAAGTCTATTACTTCGTAATTACTGCG |  |
| **TinfSat02-79** | TinfSat02-79-F  5´- TTGTAAGGTTCAAGAAAATCCC | TinfSat02-79-R  5´- CTCACTCTTACGGTTGAAACGC |
| **TinfSat03-4** | (GATA)5  5´ - GATAGATAGATAGATAGATA |  |
| **TinfSat04-1000** | TinfSat04-1000-F  5'- GATATCGAAAATTTGACACG | TinfSat04-1000-R  5'- ATGTATGTGAACAGCATAGC |
| **TinfSat05-4** | (CATA)5  5´- CATACATACATACATACATA |  |
| **TinfSat06-181** | TinfSat06-181  5´- CGGCTCAAAAACAATATTAAAGTCC |  |
| **TinfSat07-10** | TinfSat07-10  5´- rCATACTCGkrCATACTCGk |  |
| **TinfSat08-239** | TinfSat08-239-F  5´- GTTCGAGTCCATGCTCAC | TinfSat08-239-R  5´- AATTTTTAGAATAGGTTCGC |
| **TinfSat09-113** | TinfSat09-113  5´- AGAATGTAkAACTTTG |  |
| **TinfSat10-53** | TinfSat10-53-F  5´- CGGTTTTGGTTATACTATTTTTCCA | TinfSat10-53-R  5´- GAAGGGGCAAACGTGTATT |
| **TinfSat11-85** | TinfSat11-85-F  5´- CCATTTTCCTCTCAAATTGAAC | TinfSat11-85-R  5´- ATGTTAATTGCTGAATCACGC |
