# Supplementary material for: Comparative repeatome analysis on Triatoma infestans Andean and Non-Andean lineages, main vector of Chagas disease
Source: PLoS One. 2017 Jul 19;12(7):e0181635. doi: 10.1371/journal.pone.0181635 (PMC5517068; doi:10.1371/journal.pone.0181635)
Supplement: S1 Fig — (DOCX) [file pone.0181635.s003.docx]

**S1 Figure:** Aligment of the four regions of the consensus monomeric unit of the TinfSat09-113 satDNA showing internal similarities that could suggest that this satDNA is really a HOR with four subrepeats.
